# Fit Hearts, Better Outcomes? A Systematic Review and Meta-Analysis of Exercise Intensity and Peak VO_2_ in Hypertrophic Cardiomyopathy

**DOI:** 10.3390/jcm14217466

**Published:** 2025-10-22

**Authors:** Andrija Djuranovic, Jovana Ristic, Milena Antic, Nina Rajovic, Mladen Mirkovic, Djordje Batinic, Milos Maletic, Sevda Ece Kizilkilic, Victoria Zecchin Ferrara, Verica Prodanovic, Suzana Savic, Sanja Mazic, Natasa Milic

**Affiliations:** 1 Medical Department, Serbian Institute of Sport and Sports Medicine, 11000 Belgrade, Serbia; djuranovic.andrija@gmail.com (A.D.);; 2 Faculty of Medicine, University of Belgrade, 11000 Belgrade, Serbia; jovanaristic00@gmail.com (J.R.); mladenm24mirkovic@gmail.com (M.M.);; 3 Institute for Medical Statistics and Informatics, Faculty of Medicine, University of Belgrade, 11000 Belgrade, Serbia; 4 Medicine and Life Sciences, Hasselt University, 3500 Hasselt, Belgium; 5 Faculty of Medicine and Health Sciences, Ghent University, 9000 Ghent, Belgium; 6 Department of Medicine and Surgery, University of Padua, 35122 Padua, Italy; 7 Department of Internal Medicine, Faculty of Medicine Foca, University of East Sarajevo, 73300 Foca, Bosnia and Herzegovina; 8 Family Medicine, Public Health Institution “Health Center” Banja Luka, 78000 Banja Luka, Bosnia and Herzegovina; 9 Institute of Medical Physiology, Faculty of Medicine, University of Belgrade, 11000 Belgrade, Serbia; 10 Center for Sports Medicine and Exercise Therapy, Faculty of Medicine, University of Belgrade, 11000 Belgrade, Serbia

**Keywords:** training, peak oxygen consumption, ventricular tachycardia, syncope, atrial fibrillation

## Abstract

**Background:** This study aimed to systematically review and analyze the available evidence on the safety and efficacy of physical activity (PA) in patients with HCM. **Methods:** We conducted a systematic search of PubMed, Cochrane, and Web of Science databases up to March 30, 2025. Fourteen studies (4 RCTs) were included in the qualitative synthesis and ten in the quantitative synthesis, totaling 10478 patients. **Results:** The meta-analysis demonstrated a significant improvement in peak VO_2_ in the moderate intensity PA (MIPA) group, with a mean difference of 1.77 mL/kg/min (95% CI: 0.93 to 2.60, I^2^ = 38.2%, *p* = 0.19), while changes in body mass index were not significant (MD: −0.66 kg/m^2^; 95% CI: −1.77 to 0.44; I^2^ = 62%; *p* = 0.07). No significant differences were observed in the occurrence of non-sustained ventricular tachycardia (NSVT) (OR = 1.54, 95% CI: 0.93 to 2.52, I^2^ = 28.3%, *p* = 0.24), atrial fibrillation (OR = 0.89, 95% CI: 0.77 to 1.03, I^2^ = 28.6%, *p* = 0.23), or syncope (OR = 1.23, 95% CI: 0.72 to 2.10, I^2^ = 25.6%, *p* = 0.24) between the MIPA and sedentary group. Additionally, the occurrence of NSVT between the high-intensity PA and MIPA group showed no significant difference (OR = 1.19, 95% CI: 0.60 to 2.36, I^2^ = 0%, *p* = 0.99). **Conclusion:** The results suggest that regular exercise does not increase the risk of NSVT, AF, or syncope while enhancing peak VO_2_, indicating that regular exercise is safe and beneficial in HCM patients.

## 1. Introduction

Hypertrophic cardiomyopathy (HCM) is defined as left ventricular hypertrophy (LVH) developing in the absence of cardiac, systemic, or metabolic disease capable of producing the degree of hypertrophy observed in HCM. It is the most common genetic cardiomyopathy, with a prevalence of 1:200 to 1:500 [1]. HCM in adults is characterized by a left ventricular (LV) wall thickness of 15 mm or more. In individuals with a positive family history and a positive genetic test, a wall thickness of 13–14 mm can be diagnostic [2]. Genetic testing of HCM patients has revealed that pathogenic variations in genes encoding sarcomere proteins account for 30–40% of cases, with the most frequent being those in *MYBPC3*, *MYH7*, and *TNNT2.* The current utility of genetic testing is to assist in the diagnostic process and family screening [3]. Molecular and histological phenotypes of HCM, including myocyte and cardiac hypertrophy, disarray, and interstitial fibrosis, result from molecular changes in the heart that lead to the morphological and clinical manifestations of HCM. While initially the patients are minimally or asymptomatic, the most frequent symptoms include exertional dyspnea, exercise intolerance, chest pain, palpitations, pre-syncope, and syncope. The symptoms arise from morphological and hemodynamic changes, including LV diastolic dysfunction, LV outflow tract obstruction (LVOTO), an imbalance between myocardial oxygen supply and demand, and cardiac arrhythmias. Prolonged LVOTO and severe interstitial fibrosis can lead to the thinning and enlargement of the LV wall, resulting in a decline in systolic function, which may progress to heart failure with reduced ejection fraction, sometimes referred to as “burnt out HCM” [4,5]. In addition to an echocardiogram (ECHO), cardiac magnetic resonance (CMR) is recommended for a comprehensive assessment of the heart’s structural and morphological characteristics. This includes evaluating systolic and diastolic function, chamber dimensions, the thickness of all LV wall segments, the mitral valve for the presence and severity of mitral regurgitation, assessing systolic anterior motion, measuring the left ventricular outflow tract (LVOT) pressure gradient, and evaluating the presence and distribution of fibrosis/late gadolinium enhancement (LGE) [6]. Although the incidence of sudden cardiac death (SCD) is low, estimated at 0.5–1% per year, it can occur as the initial presentation of HCM, regardless of symptoms [7]. Traditionally, patients with HCM have been restricted from participating in sports due to concerns about their risk of SCD. Exercise recommendations have remained conservative, regardless of symptomatic status, cardiac morphology, risk profile, or prior surgical interventions, such as implantable cardioverter-defibrillators (ICDs) or invasive methods to reduce left ventricular outflow obstruction. Not promoting an active lifestyle and imposing restrictions has contributed, in part, to a sedentary lifestyle among HCM patients, which increases the prevalence of obesity and leads to adverse health outcomes. Over the years, data have shown more favorable outcomes for HCM patients regarding physical activity (PA), leading to a shift in the paradigm of exercise prescription for these individuals [8,9]. According to the World Obesity Atlas, projections show that if current trends persist, nearly 3 billion adults (around 50% of the world’s adult population) will be affected by overweight and obesity by 2030. Regular physical activity is crucial for preventing and managing obesity and non-communicable diseases [10]. Therefore, we conducted this systematic review and meta-analysis to investigate the safety (non-sustained ventricular tachycardia (NSVT), atrial fibrillation (AF), and syncope) and efficacy (peak oxygen consumption, VO_2_peak, and body mass index, BMI) outcomes of moderate-intensity physical activity (MIPA) compared to a sedentary lifestyle, and to compare the safety of high-intensity physical activity (HIPA) to MIPA in patients with HCM.

## 2. Methods

This systematic review and meta-analysis was conducted and reported in accordance with the recommendations of the Cochrane Collaboration [11], and the Preferred Reporting Items for Systematic Reviews and Meta-Analyses (PRISMA) guidelines [12].

### 2.1. Eligibility Criteria

Studies were included in this meta-analysis if they met the following eligibility criteria: (1) patients with HCM (phenotype-positive-genotype-negative or phenotype-negative-genotype-positive); (2) assessed any level of PA, ranging from moderate to high intensity; (3) reported at least 1 of the predefined outcomes of interest; and (4) were randomized controlled trials (RCTs) or observational studies (cross-sectional, cohort, or case–control designs).

The following were excluded: (1) duplicate publications; (2) conference abstracts; (3) scoping reviews; (4) systematic reviews; (5) narrative reviews; (6) case reports; (7) studies focusing solely on cardiomyopathies other than HCM; (8) in vitro or animal studies; and (9) studies that did not report any relevant outcomes.

### 2.2. Search Strategy and Data Extraction

We conducted a systematic search of the PubMed, Cochrane, and Web of Science databases up to 30 March 2025. The search was performed by two independent authors (A.D. and J.R.). The detailed search strategy is provided in Supplementary Table S1. No restrictions were applied regarding publication date or language. A protocol detailing the research methodology was registered in the International Prospective Register of Systematic Reviews (PROSPERO CRD42025644368) prior to the commencement of the review. All pre-specified outcomes from the registered protocol are reported in this review; additional outcomes identified during the review process were included to ensure a more comprehensive synthesis. Retrieved articles were imported into Rayyan [13], a software tool for systematic review management. Two authors (A.D. and J.R.) independently screened titles and abstracts based on predefined selection criteria. Subsequently, the same authors performed a full-text review of all eligible articles, resolving any disagreements by consensus. Authors were contacted for missing Supplementary Information. Data extraction was conducted independently by the 3 authors (A.D., J.R., and M.M.) utilizing a standardized data extraction form.

### 2.3. Effect Measures

The primary outcome was the change in peak oxygen consumption (VO_2_peak) between the moderate-intensity physical activity group (MIPA, intervention) and the sedentary group (control), both consisting of patients with HCM. Secondary outcomes included adverse events: the occurrence of non-sustained ventricular tachycardia (NSVT), atrial fibrillation (AF), and syncope. The secondary outcome also included the change in body mass index (BMI) from baseline to follow-up. Continuous outcomes (VO_2_peak and BMI) were reported as mean ± standard deviation (SD), while binary outcomes were reported using Peto’s odds ratio (OR).

### 2.4. Quality Assessment

The risk of bias in RCTs was assessed using the Revised Cochrane Risk of Bias tool (RoB 2), which encompasses 5 domains of bias. Each study was evaluated as having low risk, some concerns, or high risk of bias. Risk of bias in observational studies was evaluated with the NIH tool for pre–post studies without a control group and the Newcastle–Ottawa Scale (NOS) for case-control and cohort studies. Assessments were carried out independently by 2 reviewers (A.D. and J.R.), with any disagreements resolved by consensus.

### 2.5. Sensitivity Analysis

For meta-analyses involving more than 3 studies, a leave-one-out sensitivity analysis was performed to evaluate the robustness of the results and identify possible sources of heterogeneity.

### 2.6. Statistical Analysis

Continuous outcomes were reported as mean ± standard deviation (SD), while binary outcomes were expressed as Peto’s odds ratio (OR). Peto’s odds ratio was selected for its performance with rare events and low-event-rate studies. For studies not reporting SDs for change scores, these were estimated from 95% confidence intervals, means, number of participants, and confidence interval limits using the RevMan (Cochrane Collaboration, Copenhagen, Denmark) Calculator, which allows derivation of standard deviations from these reported summary statistics. Between-study heterogeneity was assessed using Cochran’s Q test, the I^2^ statistic, and tau-squared (τ^2^) to measure variance. An I^2^ value greater than 50% was considered indicative of substantial heterogeneity; therefore, a random-effects model was employed in such cases. All statistical analyses were conducted using R version 4.4.2 (R Foundation for Statistical Computing, Vienna, Austria) via the RStudio integrated development environment, version 2024.12.0 + 467 (RStudio, PBC, Boston, MA, USA).

## 3. Results

**Figure 1:**
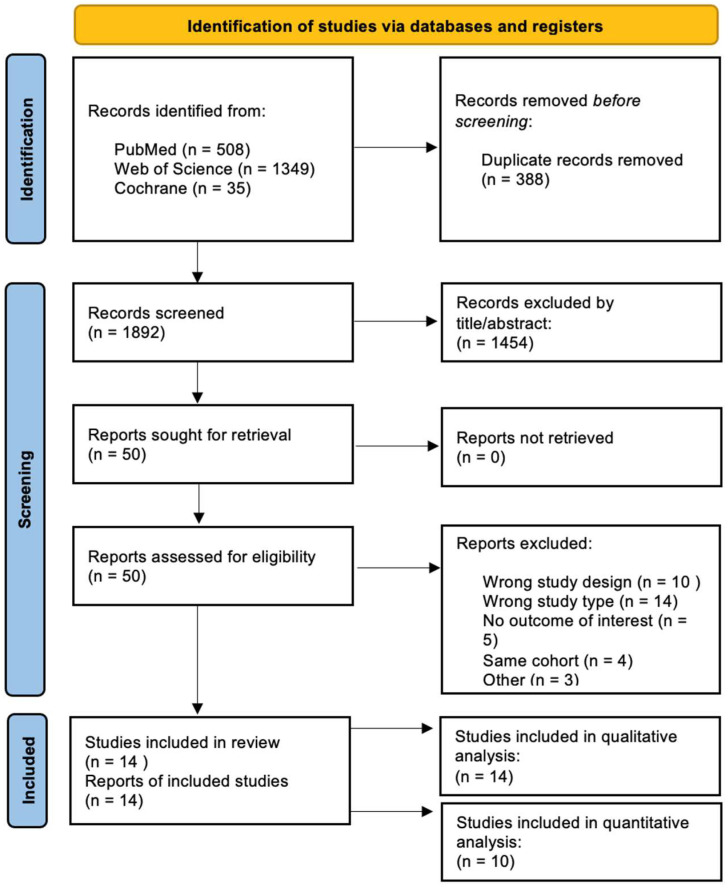
PRISMA flow diagram regarding article selection for the systematic review and meta-analysis. PRISMA = Preferred Reporting Items for Systematic Reviews and Meta-analyses.

The search strategy initially identified 1892 results from all databases. Of these, 388 duplicates were removed, and 1454 articles were excluded after reviewing the titles and abstracts. Ultimately, 50 articles underwent full-text screening. A total of 14 were included in the qualitative synthesis [14,15,16,17,18,19,20,21,22,23,24,25,26,27], and 10 of those were included in the quantitative synthesis (3 for change in VO_2_peak and BMI [14,15,26], 5 for the occurrence of AF [17,20,21,24,26], 6 for the occurrence of syncope [14,17,18,21,24,26] and 4 for the occurrence of NSVT [17,21,24,26]). The selection process is illustrated in Figure 1.

### 3.1. Characteristics of the Included Studies and the Risk of Bias

Fourteen studies were included [14,15,16,17,18,19,20,21,22,23,24,25,26,27], 4 of which were randomized controlled trials [14,15,19,26]. In total, 10,478 participants were involved (10,114 patients, excluding genotype-positive-phenotype-negative), out of which 3342 (32%) were female. A total of fifty-one participants were lost to follow-up in interventional studies. Studies have compared different levels of PA, with the majority of studies examining MIPA versus a sedentary lifestyle in patients with HCM. PA levels were defined differently among studies. RCTs categorized PA intensity, which is in accordance with intensity classifications outlined in the 2020 ESC Guidelines on sports cardiology and exercise for patients with cardiovascular disease [15]. The definition of HI and MI somewhat varies across the studies, which is explored in detail in the Supplementary Table S2. The diagnosis of HCM was based on either the European Society of Cardiology (ESC) or American Heart Association/American College of Cardiology (AHA/ACC) guidelines, with the most recent guidelines applicable at the time of publication. The characteristics of the studies are presented in Table 1 and Supplementary Table S3. Based on the RoB 2, all RCTs showed a low risk of bias (Supplementary Figure S1). Assessment of study quality for observational studies using the NIH tool and NOS indicated that five studies were of good quality and five were of fair quality (Supplementary Tables S4–S6).

### 3.2. Change in VO_2_peak

**Figure 2:**
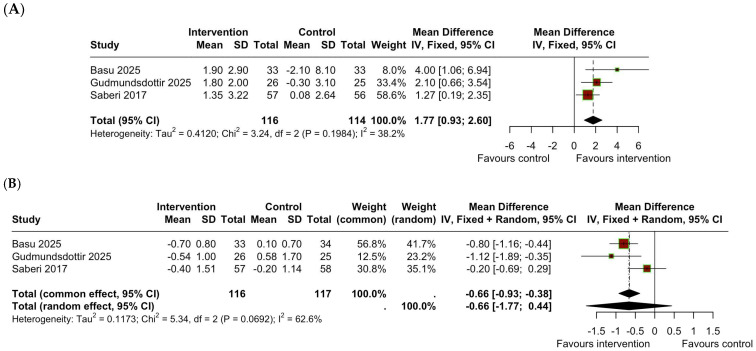
Forest plot showing the change in peak VO_2_ between the exercise and the sedentary groups (**A**) and forest plot showing the change in BMI between the exercise and the sedentary group (**B**). BMI = body mass index; IV = Inverse Variance; SD = Standard Deviation; CI = Confidence Interval.

Three RCTs evaluated the effect of PA compared to a sedentary lifestyle in patients with HCM on VO_2_peak [14,15,26]. The meta-analysis demonstrated a significant improvement in VO_2_peak in the exercise group, with a mean difference of 1.77 mL/kg/min (95% CI: 0.93 to 2.60, I2 = 38.2%, *p* = 0.19), as shown in Figure 2A.

### 3.3. Change in BMI

The effect of PA compared to a sedentary lifestyle in patients with HCM was also assessed in the 3 RCTs [14,15,26]. The meta-analysis (Figure 2B) showed no significant reduction in BMI in the exercise group, with a mean difference of −0.66 kg/m^2^ (95% CI: −1.77 to 0.44, I^2^ = 62%, *p* = 0.07).

### 3.4. Adverse Outcomes

**Figure 3:**
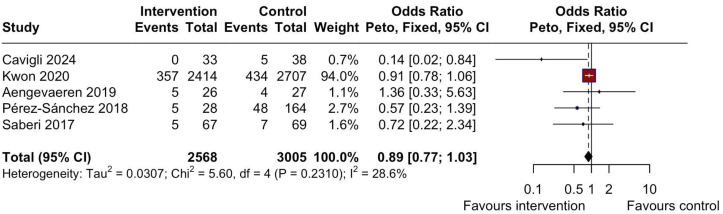
Forest plot depicting the difference in the occurrence of AF between MIPA and the sedentary control group. AF = atrial fibrillation; MIPA = moderate-intensity physical activity; CI = Confidence Interval.

**Figure 4:**
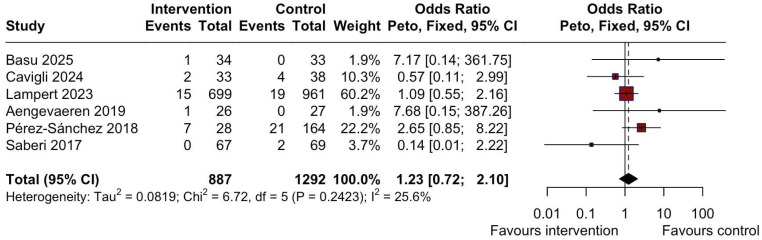
Forest plot showing the difference in the occurrence of syncope between the MIPA and the sedentary control groups. MIPA = moderate-intensity physical activity; CI = Confidence Interval.

**Figure 5:**
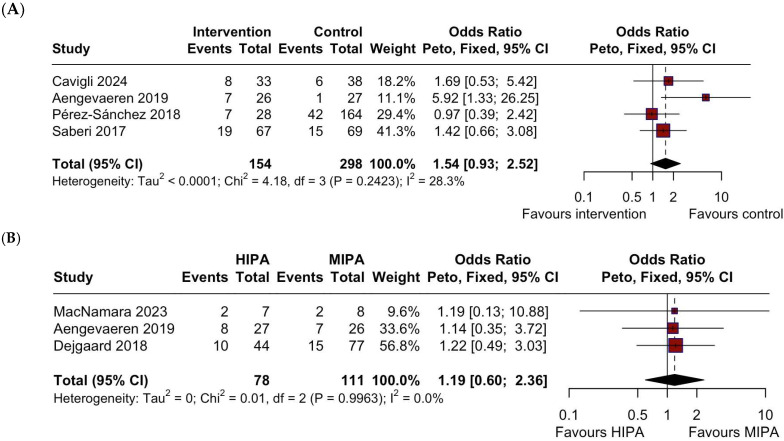
Forest plot depicting the difference in the occurrence of NSVT between (**A**) MIPA and the sedentary control group, and (**B**) HIPA and MIPA groups. HIPA, high-intensity physical activity; MIPA = moderate-intensity physical activity; NSVT = non-sustained ventricular tachycardia; CI = Confidence Interval.

No significant differences were observed in the occurrence of AF (Figure 3) (OR = 0.89, 95% CI: 0.77 to 1.03, I^2^ = 28.6%, *p* = 0.23) [17,20,21,24,26], syncope (Figure 4) (OR = 1.23, 95% CI: 0.72 to 2.10, I^2^ = 25.6%, *p* = 0.24) [14,17,18,21,24,26], or NSVT (Figure 5A) (OR = 1.54, 95% CI: 0.93 to 2.52, I^2^ = 28.3%, *p* = 0.24) [17,21,24,26] between the intervention (MIPA) and control (sedentary) groups.

Additionally, a meta-analysis of studies comparing the differences in the occurrence of NSVT between high-intensity physical activity (HIPA) and MIPA groups (Figure 5B) also showed no significant difference (OR = 1.19, 95% CI: 0.60 to 2.36, I^2^ = 0%, *p* = 0.99) [19,21,25].

### 3.5. Leave-One-Out Analysis

The included RCTs [14,15,19,26] employed diverse protocols for physical activity prescription, encompassing different modalities and varying levels of supervision. Such variations may have contributed to clinical and methodological heterogeneity. In addition, the observational studies relied on different questionnaires to quantify physical activity levels, potentially introducing recall bias (Supplementary Table S2). To evaluate the robustness of the findings and identify influential studies, leave-one-out sensitivity analyses were performed for each outcome. For AF, the overall pooled effect estimates using Peto’s method was not statistically significant (OR = 0.8871; 95% CI: 0.7657–1.0277; *p* = 0.1105), with low to moderate heterogeneity (I^2^ = 28.6%). Excluding the study by Cavigli et al. [17] reduced heterogeneity to 0%, indicating it was the primary contributor to between-study variability. Nevertheless, the overall effect estimate remained consistent across all leave-one-out iterations, showing that no single study significantly altered the direction or significance of the results (Supplementary Figure S2). For NSVT, the overall heterogeneity was similar (I^2^ = 28.3%), but excluding the study by Aengevaeren et al. [21] reduced I^2^ to 0%, highlighting its influence on variability. Still, the overall direction of the effect remained stable (Supplementary Figure S3). Regarding syncope, heterogeneity initially was 25.6%, and the omission of Saberi et al. [26] reduced it to 5.6%, indicating that this study contributed to the observed variability (Supplementary Figure S4). In all cases, the direction and significance of the pooled effect estimates remained unchanged, indicating that the meta-analysis conclusions were not unduly influenced by any single study.

## 4. Discussion

This is the first meta-analysis to evaluate the safety and efficacy of various exercise intensities in patients with HCM. This study included both RCTs and observational studies. Our results indicate that regular PA in HCM increases VO_2_peak, and is safe, as there is no difference in the occurrence of NSVT, syncope, and AF between the exercise and sedentary groups.

### 4.1. Exercise Interventions

As outlined in Table 1, different exercise protocols were prescribed in the interventional studies. The American College of Sports Medicine (ACSM) suggests the “FITT” principle (frequency, intensity, time, and type) to prescribe an exercise program [28]. In the 3 RCTs [14,15,16], exercise was prescribed 3 times per week, except by Saberi et al. [26] where the patients gradually increased the frequency, with the goal of 4 to 7 times per week. Cavigli et al. [17] increased the frequency up to 5 times per week, and Klempfner et al. [27] prescribed exercise twice per week. Intensity was determined in accordance with the classification outlined in the 2020 ESC Guidelines on sports cardiology and exercise for patients with cardiovascular disease [29]. Interventional studies mainly employed MIPA, except in studies by Basu et al. [14], Cavigli et al. [17], Mac Namara et al. [19], and Klempfner et al. [27] which included HIPA programs. Exercise sessions lasted approximately 1 hour in all studies. All interventional studies included an aerobic type of activity. Basu et al. [14], Gudmundsdottir et al. [15], and Cavigli et al. [17] also prescribed RT. Other types of training, like balance and stretching, were not described. A key challenge noted in some trials [e.g., Basu et al. [14]] was maintaining long-term exercise adherence, as benefits diminish after supervised programs end, emphasizing the need for strategies to support ongoing engagement.

### 4.2. Safety

In the era of modern treatment and appropriate risk stratification, the SCD rates in HCM are low, at approximately 0.32% per year (data varies worldwide by region) [30]. Although SCD is rare in HCM patients, it remains the most serious complication because it can occur in asymptomatic individuals. The ACC/AHA and ESC currently use 2 risk scores for patients aged ≥16 years. Both calculators include parameters such as age, maximal LV thickness, LA size, maximal LVOT pressure gradient, family history of SCD, NSVT, and unexplained syncope. The primary differences between the two are the 3 non-invasive markers obtained from ECHO and CMR, which are LV apical aneurysm, LV systolic dysfunction, and LGE on CMR, as outlined in the ACC/AHA guidelines [31].

This study analyzed data from a total of 10,478 patients, 3342 female (10,114 patients excluding genotype-positive-phenotype-negative individuals). Patient characteristics are shown in detail in Table 1. RCTs [14,15,19,26] included patients aged approximately 40 to 60 years. HCM patients with “high-risk” characteristics were excluded, such as LVOT ≥ 50 mmHg or medically refractory LVOT, EF ≤ 35%, exercise-induced arrhythmia, and syncope [1]. RCTs also excluded advanced symptomatic patients with heart failure and/or angina (NYHA III-IV, CCS III-IV), and per several other criteria. RCTs also included patients with an implanted ICD, who could be categorized as “high-risk”. Some interventional studies also included patients with comorbid conditions such as controlled hypertension, stable coronary artery disease, diabetes, lung disease, hypercholesterolemia, prior TIA or stroke, and renal failure (Supplementary Table S7). Patients continued their treatment throughout the study duration, which primarily consisted of beta blockers, calcium channel blockers, other antihypertensive agents, antiplatelet therapy, and statins. No interventional study reported the correlation between comorbidities and any of the adverse outcomes.

When comparing HIPA with MIPA or MIPA with sedentary individuals, the results suggest that exercise does not significantly increase the risk of NSVT or syncope. None of the patients in the 4 RCTs [14,15,19,26] experienced death during the exercise intervention. In a study by Basu et al. [14], 1 patient experienced asystole during exercise, while another experienced VT in a sedentary group. Furthermore, 6 patients from one group and 4 from another underwent ICD implantation due to the perceived increased risk of SCD. Gudmunsdottir et al. [15] did not report any serious adverse events, and the patients in the study by Saberi et al. [26] did not experience sustained VT, sudden cardiac arrest (SCA), or ICD shock. MacNamara et al. [19] report that two nsVT episodes, 1 in each MIPA and HIPA group, were registered using an implantable loop recorder independent of exercise and were not detected again in those participants. In the same study, over a 5-month period, no syncope, sustained VT, VF, or SCA were reported.

Other interventional studies prescribed PA, albeit without any sedentary controls. Cavigli et al. [17] and Sweeting et al. [23] reported that there were no major adverse events in any of the groups (syncope, ICD shock, aborted SCD, or death). Wasserstrum et al. [22] describe 1 NSVT episode during exercise in 1 of forty-five patients, with no significant adverse events. We would like to highlight the study by Klempfner et al. [27], which enrolled 20 HCM patients who were symptomatic despite receiving optimal medical therapy (LVEF 53 ± 15%, NYHA II-III). Notably, reduced LVEF was present in 5 patients (31 ± 6.5%), grade 2–3 diastolic dysfunction was found in 5 patients, 6 patients had an LVOT gradient at rest of 51 ± 24 mmHg, and 6 had an ICD implanted. The intervention included a supervised MIPA to HIPA program. These moderate-risk symptomatic HCM patients demonstrated significant functional improvement with no adverse events during the study period and the following 12 months (VT sustained, ICD shock, clinical deterioration). The data from the UK Pathology Registry supports these findings [7], as it reports that out of 194 cases with HCM, 150 (77%) individuals died at rest or during daily activities, 26 (13%) died during sleep, while 20 (10%) individuals were recreational or competitive athletes. Autopsy data from the US also suggest that SCD occurred predominantly during sedentary or mild activities (66%), and 32% while sleeping [32]. In contrast to this data, a study by Link et al. [33] that assessed 71 patients with HCM and implanted ICDs revealed that 57% of VF and 45% of VT episodes were associated with at least MIPA. It is important to highlight that in this study, patients were at a “high risk,” given the ICD implantation in secondary or primary SCD prevention and their morphological characteristics. Geographical disparities do exist, as HCM has been reported as the leading cause of SCD in young individuals in North America [34]. However, the data from the UK suggests that HCM (4%) is the second leading cause of SCD in myocardial disease, following arrhythmogenic cardiomyopathy (5%) [35].

AF occurs during the course of HCM, with some authors reporting its occurrence in up to 25% of HCM patients. It is generally attributed to hemodynamic factors such as diastolic dysfunction and LVOTO, which lead to the progressive LA enlargement commonly observed in patients with HCM [36]. AF is associated with significant morbidity, impaired quality of life [1], and a substantial risk of thromboembolic (TE) and stroke independent of the CHA2DS2-VASc score [37]. There is some evidence that an increase in LA size closely correlates with TE complications [38]. Current guidelines recommend annual screening for AF during the initial evaluation and suggest the use of extended ambulatory monitoring in patients with HCM who are considered to be at high risk for developing AF. If documented, an appropriate rhythm or rate control treatment strategy should be considered based on several factors [1]. Our data shows that regular exercise does not pose an increased risk of AF occurrence in HCM patients. A cohort of 14 patients participating in intense or competitive exercise in a study by Perez-Sanchez et al. [24] showed that only AF was recorded in 2 patients. A large nationwide cohort analyzed by Kwon et al. [20], which included 7666 patients, revealed no difference in AF occurrence between the least and most active individuals. Data regarding AF and regular exercise in healthy individuals is somewhat conflicting. This suggests a lower incidence of AF in physically active individuals compared to those who are sedentary. However, prolonged periods of vigorous exercise (over 9.5 h per week) may increase the risk of AF, indicating a U-shaped relationship [39]. However, that level of PA is not necessary for optimal health benefits [40]. Nonetheless, data from athletes imply that the relationship is more complex than just training volume and intensity. Other factors can contribute to AF risk, including genetic characteristics, atrial ectopy, increased vagal tone, changes in electrolytes, LA dilatation, and fibrosis [41].

The safety profile presented in this meta-analysis primarily applies to patients with low- and moderate-risk HCM. High-risk patients (e.g., those with ICDs for secondary prevention) might have a different risk-benefit ratio, and exercise recommendations for this group require extreme caution and personalized expert assessment.

### 4.3. Benefits of Exercise

Regular exercise is associated with numerous health benefits [42]. Cardiorespiratory fitness (CRF) is clearly linked to favorable health outcomes, as being unfit poses a significant risk of all-cause mortality, as demonstrated in a study of 750,000 veterans [43]. VO_2_peak is considered an indicator of CRF. It can be measured directly during cardiopulmonary exercise testing (CPET) and demonstrates an inverse correlation with cardiovascular mortality, all-cause mortality, and frailty throughout one’s lifetime [44,45]. Different types of exercise are necessary to gain significant health benefits, including various intensities of aerobic and anaerobic training, resistance training (RT), and balance exercises [40]. HCM patients with a VO_2_peak < 18 mL/kg/min had a significantly lower rate of survival free from death and severe symptoms, compared to those with VO_2_peak > 18 mL/kg/min, as well as those who exhibited a VO_2_peak < 60% of the predicted value [46]. These data emphasize the significance of CPET in evaluating HCM, mainly when used in conjunction with stress ECHO [47,48]. Results from 4 RCTs [14,15,19,26] in our analysis indicate that exercise enhances VO_2_peak. It is essential to note that all these studies included CPET in their protocol, which enabled the direct measurement of VO_2_peak. Both studies by Basu et al. [14] and Gudmundsdottir et al. [15] were supervised and lasted 12 weeks. The most significant effect on VO_2_peak increase is noted in the study by Basu et al. [14], which is somewhat expected, as the intervention involved HIPA and included both aerobic training and RT. MacNamara et al. [19] compared the effects of HIPA and MIPA programs. The HIPA group experienced a slightly (though not statistically significant) greater increase in VO_2_peak (+1.1 mL/kg/min) than the MIPA group, yet both interventions showed a significant increase in VO_2_peak from baseline. Gudmundsdottir et al. [15] and Saberi et al. [26] employed the MIPA program. However, Saberi et al. [26] did not include RT or any burst-type activities in their MIPA program; the exercise was unsupervised, and after 16 weeks, the lowest effect was observed in their group.

In a study by Cui et al., which investigated 752 patients, obesity was found to be the cause of low VO_2_peak in 48.2% of patients [49]. In another retrospective study, it is suggested that an increased BMI is associated with a higher prevalence of dyspnea, lower VO_2_peak (mL/kg/min), and shorter CPET duration, implying that weight loss may improve exercise capacity [50]. In our study, no significant reduction in BMI was observed during the study period after performing a meta-analysis of all 3 RCTs [14,15,26]. However, both Basu et al. [14] and Gudmundsdottir et al. [15] demonstrate a significant reduction in BMI. This may be due to the effect of HIPA employed by Basu et al. [14], as well as the fact that both studies included RT. The limitations of BMI in the diagnosis of obesity, such as the lack of insight into body composition, body fat distribution, etc., are noted. Nevertheless, BMI was used in the majority of the studies, and it does correlate well with health risks, given that none of the participants were athletes for whom BMI is an unreliable parameter given the high muscle mass [51]. Also, data on nutritional intake is not provided in any of the studies, and it would be necessary to estimate the effect of exercise on BMI reduction more accurately.

Interpreting the increase in VO_2_peak expressed in mL/kg/min (relative) alongside a simultaneous decrease in BMI (BM) should be approached with caution, as, in 2 individuals with the same absolute VO_2_ value (mL/min), the relative VO_2_ value will be lower for the one with a higher BM. Nonetheless, this should not diminish the numerous positive effects of exercise on reducing body mass and improving various health parameters. This is also reflected in the enhancement of certain Quality of Life (QOL) domains as evidenced by different questionnaires used in the studies. The significant improvement in VO_2_peak despite a non-significant change in BMI suggests that the benefits of exercise in HCM are driven by improvements in CRF and likely cardiac/output and peripheral muscle adaptations, not merely weight loss.

Basu et al. [14] make an important point regarding exercise adherence. In their study, some patients were reevaluated after 6 months, and a return to their usual baseline activity was observed, along with the loss of most of the initially noted benefits, suggesting that remote programs could be an option to improve adherence. Therefore, in addition to using the “FITT” principle to prescribe an exercise program for HCM patients, adherence is equally important for achieving long-term benefits.

In the modern era, HCM is a declining cause of SCD in young people, which is attributed to improved family screening, more accessible ECHO, a better understanding of risk stratification, optimal primary and secondary SCD prevention, and enhanced treatment options [52]. Considering the increased prevalence of obesity in HCM patients [8], and the fact that abdominal obesity can be observed as a central node in the most common age-related chronic diseases [53]. Lifestyle factors, including sedentary behavior, excessive calorie intake, and poor diet, must be addressed to improve outcomes in patients with HCM. As stated in both the latest AHA/ACC and ESC recommendations, engaging in mild to moderate-intensity recreational exercise is beneficial for enhancing CRF, QOL, and overall health [1,6]. The best approach would be to recommend exercise based on a comprehensive assessment and individual risk evaluation. At the same time, engaging in vigorous recreational activities and competitive sports requires collaborative decision-making with a professional who evaluates potential benefits against risks [54]. According to the 2024 guidelines, it is not recommended to universally restrict most patients with HCM from vigorous PA or competitive sports [1].

### 4.4. Strengths

This meta-analysis is the first one to evaluate the safety and efficacy of various exercise intensities in patients with HCM. The studies included show a low risk of bias. The leave-one-out sensitivity analysis remains consistent. Additionally, low heterogeneity is observed.

### 4.5. Limitations

The authors also acknowledge several limitations. The primary limitation is that the findings pool somewhat disparate interventions and control conditions. The definition of HIPA and MIPA varies across studies (Supplementary Table S2). The operational definition of ‘sedentary’ control groups also varied across studies and may not reflect actual inactivity, which could diminish the observed effect sizes. This meta-analysis includes a small number of RCTs; therefore, there is insufficient data for subgroup analysis. The studies primarily involved low-risk patients aged approximately 40 to 60 years, more than 65% male. Data on nutritional intake should be provided to accurately assess the changes in BMI. There are not enough studies with long-term follow-up to evaluate the extended safety and benefits of exercise in HCM. Additionally, observational studies categorize PA intensity in various ways. We were unable to conduct meta-regression or detailed subgroup analyses (e.g., by genotype, obstruction status) because of the limited number of studies and the absence of reporting on individual patient data, which could be important effect modifiers. The results are most applicable to middle-aged (40–60 years), predominantly male, low-to-moderate-risk patients, which limits generalizability to other demographics.

## 5. Conclusions

This study aimed to evaluate the effect of different intensities of physical activity on patients with HCM. We conducted a systematic review and meta-analysis of RCTs and observational studies to assess the safety and efficacy of PA in these patients. The results suggest that in low- to moderate-risk patients with HCM, supervised exercise training at moderate to high intensity significantly enhances cardiorespiratory fitness without raising the short-term risk of life-threatening arrhythmias or syncope. Clinicians should include individualized exercise prescriptions, based on comprehensive risk stratification, in the management plan for HCM patients. Further RCTs involving younger patients, more female patients, including those who are symptomatic and those at increased risk for SCD, with various exercise modalities and more extended follow-up periods, are necessary to evaluate the long-term safety and benefits of regular exercise.

## Figures and Tables

**Table 1:** Characteristics of the included studies.

Author and Publication Year	Design	Participant Number	Intervention	Control	Intervention Duration	Key Outcomes
Basu et al. 2025 [14], ^a^	RCT	*n* = 80	HI exercise program 3 h/week: −2 h/week supervised + 1 h/week home-based Aerobic + RT, 70 to 85% calculated HRR	Usual care	12 weeks	After 12 weeks, participants with HCM who performed HI exercise vs. usual care, increased their VO_2_peak (+1.9 ± 2.9 mL/kg/min—exercise group and −2.1 ± 8.1 mL/kg/min -usual care group), decreased BMI (−0.7 ± 0.8 mL/kg/min—exercise group and +0.1 ± 0.7 mL/kg/min -usual care group), with no increase in arrhythmias, and one syncope episode in the exercise group.
Gudmundsdottir et al. 2025 [15], ^a^	RCT	*n* = 59	Supervised MI exercise programme 3 h/week −60% maximal work capacity −12–14 RPE—aerobic and RT	Usual activity	12 weeks	In patients with HCM without LVOT obstruction, a 12-week supervised MI training intervention compared with usual activity increased VO_2_peak ((+1.8 ± 2.0 mL/kg/min—exercise group and −0.3 ± 3.1 mL/kg/min -usual care group) and decreased BMI (−0.54 ± 1.0 mL/kg/min—exercise group and +0.58 ± 1.7 mL/kg/min -usual care group).
Hassanzada et al. 2024 [16]	Cross-sectional	*n* = 133	N/A	N/A	N/A Follow-up for 8.8 (4.3–16.5) y	In truncating *MYBPC3* founder variant carriers, overall PA and high-static exercise are not associated with an increased risk of MCE and cardiomyopathy penetrance. Those who participated in the highest quartile of high-dynamic exercise had an increased risk of MVA.
Cavigli et al. 2024 [17], ^a^	Cohort	*n* = 71	Unsupervised-advice only -Personalized, tailored according to the CPET (aerobic MI, around VT1) −2 h/week and increased to 3–5 h/week -RT in non-obstructive patients, 40–70% 1RM.	N/A	N/A Reassessment in 6–12 months, followed up for max 3 years; 13 evaluated after 24 ± 12 months	Patients with HCM practicing regular MI aerobic exercise have a better functional capacity in the absence of relevant events vs. sedentary patients. A sedentary lifestyle led to a deterioration of cardiopulmonary functional capacity and fitness.
Lampert et al. 2023 [18], ^a^	Cohort	*n* = 1660	Self-reported PA in the past year (Minnesota Leisure Time Activity Questionnaire), classified according to the 2011 Compendium of Physical Activities	Sedentary	36 months (outcome surveys every 6 months)	Among individuals with HCM or those who are genotype positive/phenotype negative and are treated in experienced centers, those exercising vigorously did not experience a higher rate of death or life-threatening arrhythmias than those exercising moderately or those who were sedentary (syncope episodes: 15 in the intervention group, 19 in the control group).
Mac Namara et al. 2023 [19], ^a^	RCT	*n* = 22	Randomized (LVOT 30 mmHg cutoff): 5 months MI (*n* = 9 completed) or 1 month MI + 4 months HI (*n* = 8 completed) -Intensity based on CPET.	MI	5 months	In HCM patients, exercise training, both HI and MI, improved fitness without a clear superiority of either. Exercise training resulted in salutary peripheral and cardiac adaptations. No serious adverse events occurred (NSVT episodes: 2 in the intervention group, 2 in the control group).
Kwon et al. 2021 [20], ^a^	Cross-sectional	*n* = 7666	7-day recall questionnaire	N/A	N/A	MI to vigorous- intensity PA, in a middle- aged population of patients with HCM, was associated with progressive reduction in all- cause and cardiovascular mortality. AF episodes: 357 in the intervention group, 434 in the control group.
Aengevaeren et al. 2019 [21], ^a^	Cross-sectional	*n* = 102	Questionnaire—lifelong PA per decade	N/A	N/A	Lifelong physical activity volumes are not associated with genotype-to-phenotype transition in HCM gene carriers. AF episodes: 5 in the intervention group, 4 in the control group; syncope episodes: 1 in the intervention group, 0 in the control group; For HI vs. MI, NSVT episodes: 8 in the intervention group, 7 in the control group. For MI vs. sedentary, NSVT episodes: 7 in the intervention group, 1 in the control group.
Wasserstrum et al. 2019 [22]	Pre-post	*n* = 45	N/A (retrospective evaluation of the improvement in exercise capacity after cardiac rehabilitation)	Participants serving as their own controls	N/A	Exercise rehabilitation appears to be a suitable and safe option in HCM. It primarily benefits patients with significant functional limitations. No significant arrhythmias or adverse events were recorded during participation
Sweeting et al. 2018. [23]	Pre-post	*n* = 25	Face-to-face motivational interview (based on principles of control theory)	Participants serving as their own controls	12 w	A 12-week control theory-based intervention to increase physical activity in HCM patients led to significant improvement in physical quality of life and self-efficacy, and fewer barriers were identified.
Perez Sanchez et al. 2018 [24], ^a^	Cohort	*n* = 272	PA 2 years before the time of diagnosis in unaffected carriers or to the time of first evaluation in unaffected carriers. “Typical week” PA level is classified according to hours per week and type of activity, including physically demanding jobs	Sedentary	5.5 ± 3.3 years follow-up	Men and athletes who are carriers of sarcomeric mutations are diagnosed earlier than women and sedentary individuals. Sex, hypertension, and the degree of PA were not significantly associated with the severity of LVH. AF episodes: 5 in the intervention group, 48 in the control group; syncope episodes: 7 in the intervention group, 21 in the control group; NSVT episodes: 7 in the intervention group, 42 in the control group).
Dejgaard et al. 2018 [25], ^a^	Cross sectional	*n* = 187	Lifelong PA (since the age of 6)	N/A	N/A	Increased lifetime vigorous exercise was associated with larger LV volumes in HCM, but correlated to LV mass only in Genotype+ LVH-. Vigorous exercise was associated with favorable diastolic function in HCM LVH+, and was not related to significant VA (NSVT episodes: 10 in the intervention group, 15 in the control group).
Saberi et al. 2017 [26], ^a^	RCT	*n* = 136	Unsupervised structured MI exercise programme according to the CPET: at least 3x/week and 20 min/session. -HR at 60% HRR -RPE 11–14 -Increasing gradually 5–10 min up to 60 min, 4–7x/week at 70% HRR. -Aerobic: cycling, walk-jog, elliptical. -No RT or burst-type activity	Usual activity	16 weeks	After 16 weeks, MI exercise compared with usual activity resulted in an increase in VO_2_peak (+1.35 ± 3.22 mL/kg/min—exercise group and +0.8 ± 2.64 mL/kg/min -usual care group), slight reduction in BMI (−0.4 ± 1.51 mL/kg/min—exercise group and −0.2 ± 1.14 mL/kg/min -usual care group). There were two syncope episodes in the control group (0 in the exercise group), 5 AF episodes in the exercise group vs. 7 in the control group, 19 NSVT episodes in the exercise group vs. 15 in the control group, and no occurrences of sustained VA, SCA, appropriate defibrillator shock, or death in either group.
Klempfner et al. 2015 [27]	Pre-post	*n* = 20	Supervised, aerobic, intensity according to the EST, gradually increased from 50% to 85% of the HRR (RPE 13–15), 2 h/week (ICD patients were limited to 20 bpm below therapy threshold)	Participants serving as their own controls	Not stated, patients completed an average of 41 ± 8 h of training	Patients with HCM who remain symptomatic despite medical therapy may achieve considerable functional improvement through a supervised ET program

^a^ Studies included in the quantitative analysis. Abbreviations: CPET = cardiopulmonary exercise test; EST = exercise stress test; ET= exercise training; HCM = hypertrophic cardiomyopathy; HI = high intensity; HRR = heart rate reserve; ICD, implantable Cardioverter-Defibrillator; LVOT = left ventricular outflow tract; MI = moderate intensity; MVA = major ventricular arrhythmia; N/A = not applicable; NSVT = non-sustained ventricular tachycardia; PA = physical activity; RCT = randomized controlled trial; RM = repetition maximum; RPE = rating of perceived exertion; RT = resistance training; SCA = sudden cardiac arrest; VA = ventricular arrhythmia; VO_2_peak = peak oxygen consumption; VT1 = first ventilatory threshold.

## Data Availability

All the available data are presented in the manuscript and the Supplementary Data.

## Supplementary Materials

The following supporting information can be downloaded at: https://www.mdpi.com/article/10.3390/jcm14217466/s1, Table S1: Search Strategy; Table S2: Detailed exercise intervention and intensity in the studies; Table S3: Detailed participant inclusion, exclusion criteria and evaluation protocol; Table S4. Newcastle-Ottawa quality assessment scale for cohort studies; Table S5. Newcastle-Ottawa quality assessment scale for cross-sectional studies; Table S6. Quality assessment using NIH Quality assessment Tool for Before-After (Pre-Post) studies with no control group; Table S7: Comorbid conditions of HCM patients in the interventional studies; Figure S1: Risk of bias assessment; Figure S2: Sensitivity analysis for the difference in the occurrence of AF between the MIPA and the sedentary control group; Figure S3: Sensitivity analysis for the difference in the occurrence of NSVT between the MIPA and the sedentary control group; Figure S4: Sensitivity analysis for the difference in the occurrence of syncope between MIPA and sedentary control group.
