# Supplementary material for: Fit Hearts, Better Outcomes? A Systematic Review and Meta-Analysis of Exercise Intensity and Peak VO2 in Hypertrophic Cardiomyopathy
Source: J Clin Med. 2025 Oct 22;14(21):7466. doi: 10.3390/jcm14217466 (PMC12610065; doi:10.3390/jcm14217466)
Supplement: Supplementary file 1 [file jcm-14-07466-s001.zip › jcm-3870221-supplementary.pdf]

Supplementary Material

Table S1. Search Strategy

Concept	Search Terms (Title/Abstract)
Population	"hypertrophic cardiomyopathy"[Title/Abstract]<br>"cardiac hypertrophy"[Title/Abstract]
Intervention	"exercise training"[Title/Abstract]<br>"physical training"[Title/Abstract]<br>"exercise therapy"[Title/Abstract]<br>"aerobic exercise"[Title/Abstract]<br>"resistance training"[Title/Abstract]<br>"cardiac rehabilitation"[Title/Abstract]<br>"physical activity"[Title/Abstract]<br>"structured exercise"[Title/Abstract]<br>"exercise intervention"[Title/Abstract]
Boolean Combination	("hypertrophic cardiomyopathy"[Title/Abstract] OR "cardiac hypertrophy"[Title/Abstract]) AND ("exercise training"[Title/Abstract] OR "physical training"[Title/Abstract] OR "exercise therapy"[Title/Abstract] OR "aerobic exercise"[Title/Abstract] OR "resistance training"[Title/Abstract] OR "cardiac rehabilitation"[Title/Abstract] OR "physical activity"[Title/Abstract] OR "structured exercise"[Title/Abstract] OR "exercise intervention"[Title/Abstract])

Table S2. Detailed exercise intervention and intensity in the studies

Author and publication year	Type of study	Intervention	Intervention duration	PA intensity
Basu et al. 2025 [14][a]	RCT	HI exercise program 3h/week:<br>• -2h/week supervised + 1h/week home-based<br>• -Aerobic + RT, 70 to 85% calculated HRR<br><br>(34/40 completed)	12 weeks	HI
Gudmundsdottir et al. 2025 [15][a]	RCT	-Supervised MI exercise programme 3h/week<br>-60% maximal work capacity<br>-12-14 RPE - aerobic and RT<br><br>(26/29 completed)	12 weeks	MI
Hassanzada et al. 2024 [16]	Cross-sectional	N/A	N/A<br><br>Follow-up for 8.8 (4.3-16.5) y	According to the Compendium of Physical Activities, grouped across quartiles
Cavigli et al. 2024 [17][a]	Cohort	-Unsupervised-advice only<br>-Personalized, tailored according to the CPET (aerobic MI, around VT1)<br>-2h/week and increased to 3- 5h/week<br>-RT in non-obstructive patients, 40-70% 1RM.	N/A<br><br>Reassessment in 6-12 months, followed up for max 3 years;13 evaluated after 24±12 months	MI as defined by the authors<br><br>MI according to VT1, but 70% of 1RM can be classified as HI RT, according to the 2020 ESC Guidelines on sports cardiology (Pelliccia et al.)
Lampert et al. 2023 [18][a]	Cohort	-Self-reported PA in the past year (Minnesota Leisure Time Activity Questionnaire), classified according to the 2011 Compendium of Physical Activities	36 months (outcome surveys every 6 months)	Not meeting criteria as sedentary
Mac Namara et al. 2023 [19][a]	RCT	-Randomized (LVOT 30 mmHg cutoff): 5 months MI (n=9 completed) or 1 month MI + 4 months HI (n=8 completed)<br>-Intensity based on CPET.	5 months	HI
Kwon et al. 2021 [20][a]	Cross-sectional	7-day recall questionnaire	N/A	According to the Compendium of Physical Activities, grouped across tertiles

Aengevaeren et al. 2019 [21][a]	Cross-sectional	Questionnaire - lifelong PA per decade	N/A	According to the Compendium of Physical Activities, grouped across tertiles
Wasserstrum et al. 2019 [22]	Pre-post	N/A<br><br>(retrospective evaluation of the improvement in exercise capacity after cardiac rehabilitation)	N/A	MI
Sweeting et al. 2018. [23]	Pre-post	Face-to-face motivational interview (based on principles of control theory)	12w	Recorded minutes per week of sedentary, light, moderate, vigorous, and moderate-vigorous activity
Perez Sanchez et al. 2018 [24]a	Cohort	PA 2 years before the time of diagnosis in unaffected carriers or to the time of first evaluation in unaffected carriers. "Typical week"<br><br>PA level is classified according to hours per week and type of activity, including physically demanding jobs	5.5±3.3 years follow-up	Moderate and intense
Dejgaard et al. 2018 [25][a]	Cross sectional	Lifelong PA<br>(since the age of 6)	N/A	Rated according to the Compendium of Physical Activities
Saberi et al. 2017 [26][a]	RCT	-Unsupervised structured MI exercise programme according to the CPET: minimum 3x/week and 20 min/session.<br>-HR at 60% HRR<br>-RPE 11-14<br>-Increasing gradually 5-10min up to 60min, 4-7x/week at 70% HRR.<br>-Aerobic: cycling, walk-jog, elliptical. -<br><br>No RT or burst-type activity<br><br>(57/67 completed)	16 weeks	MI
Klempfner et al. 2015 [27]	Pre-post	Supervised, aerobic, intensity according to the EST, gradually increased from 50% to 85% of the HRR (RPE 13-15), 2h/week<br><br>(ICD patients were limited to 20 bpm below therapy threshold)	Not stated, patients completed an average of 41±8 hours of training	Intensity not defined by authors<br><br>MI to HI: 50-69% HRR can be classified as MI aerobic; 70-85% HRR and RPE 15-16 can be classified as HI aerobic, according to the 2020 ESC Guidelines on sports cardiology (Pelliccia et al.)

[a] Studies included in the quantitative analysis. Abbreviations: bpm=beats per minute; CIED=cardiac implantable electronic device; CPET=cardiopulmonary exercise test; EST=exercise stress test; HCM=hypertrophic cardiomyopathy; HI=high intensity; HIPA=high-intensity physical activity; HRR=heart rate reserve; implantable Cardioverter-Defibrillator; MI=moderate intensity; MIPA=moderate-intensity physical activity; N/A=not applicable; PA=physical activity; RCT=randomized controlled trial; RM=repetition maximum; RPE=rating of perceived exertion; RT=resistance training; =first ventilatory threshold.

Table S3. Detailed participant inclusion, exclusion criteria and evaluation protocol

Author and publication year	Type of study	Participants (inclusion criteria)	Age and participant number	Exclusion criteria	Examination protocol
Basu et al. 2025 [14][a]	RCT	-16-80 years<br><br>- HCM diagnosis (LVWT ≥15mm in the absence of abnormal loading conditions), NYHA I-II<br><br>- Able to exercise	n=80 (13 female)<br><br>Exercise (n=40): 48±7.9 years<br><br>Usual care (n=40): 44±8.9 years	- Competitive athletes<br>- Previous exercise-induced syncope<br>-uncontrolled VA,<br>- LVEF<35%, LVOT ≥ 50 mmHg<br>- <3 months to planned CIED implantation<br>- Known CAD (>50% lesion, previous PCI/CABG)<br>- Exercise limited by non-cardiac cause<br>- Renal failure<br>- Phenocopies of HCM<br>- Pregnancy	Baseline and after 12 weeks:<br>-Physical<br>-ECG<br>-Biomarkers<br>-ECHO<br>-CPET<br>-48h ECG Holter<br>-QoL<br>-CMR (only baseline)
Gudmundsdottir et al. 2025 [15][a]	RCT	- ≥18 years<br><br>-HCM (LVWT≥15mm in the absence of other LV causes or LVWT ≥13mm and 1st degree relative carrying definite or likely disease-causing variant)	n=59<br>(16 female)<br><br>Exercise (n=29): 56.3±11.3 years<br><br>Usual activity (n=30): 59.9±12.9 years	- Peak LVOT ≥ 30 mmHg at rest or Valsalva (or ≥ 50 mmHg immediately after exercise)<br>-History of exercise-related syncope or VA in the last year<br>-Severe angina CCS III, IV,<br>-Severe valvular disfunction,<br>-<3m after septal reduction,<br>-Severe HTA<br>-Patients already on MI or HI exercise >1h/week<br>-Changes in medical therapy that could affect exercise capacity/hemodynamics<br>-Pregnancy/planned pregnancy<br>- Other conditions that could interfere with the participation	Baseline and after 12 weeks:<br>-Physical<br>-Biomarkers<br>-ECHO<br>-CPET<br>-QoL<br>-right heart catheterization at rest and during exercise
Hassanzada et al. 2024 [16]	Cross-sectional	-*MYBPC3* carriers (97/188 with HCM diagnosis at baseline)	n=133 with HCM diagnosis by the end of the follow-up<br><br>(80 female)<br><br>43±15 years	N/A	-Interview: PA since the age of 10<br>-Composite of malignant VA (VT or VF sustained)<br>-HF (hospitalizations or transplantations) and SRT
Cavigli et al. 2024 [17][a]	Cohort	-18-55 years<br><br>-HCM (increased LV thickness not explained by abnormal loading conditions)	n=71<br>(7 female)<br><br>Physically active (n=33): 39±14 years	-NYHA III, IV<br>-Cardiac or noncardiac causes of functional limitations<br>-SRT<br>-ICD in the past 3	Baseline and after 12 weeks:<br>-Physical<br>-Training history<br>-ECG<br>-48h ECG Holter

			Sedentary (n=38): 38±14 years	months<br>-Acute HF or hospitalization in the past 3 months<br>-Severe ventricular dysfunction<br>-Changes in therapy in the past 3 months<br>-LGE>15%<br>-Pregnancy	-Biomarkers<br>-ECHO<br>-CPET<br>- QoL
Lampert et al. 2023 [18][a]	Cohort	-8-60 years<br><br>-Overt HCM (phenotype positive), or genotype positive/phenotype negative status	n= 1660 (664 female); 1534 phenotype-positive<br><br>Nonvigorous sedentary (n=252):41.5±13.8 years<br><br>Moderate (n=709): 40.2±13.9 years<br><br>Vigorous (n=699): 36.1±15.3 years	-Conditions precluding vigorous exercise (NYHA III or IV, other advanced HCM symptoms, non-HCM related conditions)<br>-Other causes of LVH (systemic, infiltrative)	Minnesota Leisure Time Activity Questionnaire
Mac Namara et al. 2023 [19][a]	RCT	-18-65 years<br><br>-HCM (LV hypertrophy with ED wall thickness ≥15 mm or wall thickness 13-15mm with family history of HCM or positive genetic test in the absence of or out of proportion to systemic disease that can cause LVH),	n=22 (5 female)<br><br>MI group: 52.3±6.7<br><br>HI group: 42.0 ±7. 8	-Exercise-induced arrhythmias<br>-Resting LVOT ≥ 50 mmHg<br>-SRT<br>-Pregnancy<br>-NYHA IV<br>-Hypotensive response to exercise (≥20mm Hg)<br>-LV systolic dysfunction, prior MI or stroke<br>-Inability to exercise	Baseline and after 5 months:<br>-ECHO<br>-CPET<br>-QoL
Kwon et al. 2021 [20][a]	Cross-sectional	-HCM under diagnostic code I42.1 or I42.2	n=7666 (2214 female)<br><br>"Mean age around 59.5 years"	-Patients not registered as HCM under diagnostic code I42.1 or I42.2<br>-Those who did not undergo health check-ups within 1 year after the diagnosis of HCM<br>-Who responded "no physical activity at all"	Korean National Health Insurance Service database: individuals with HCM and non-zero PA
Aengevaeren et al. 2019 [21][a]	Cross-sectional	-HCM (LVH ≥15mm or ≥13mm with gene mutation and/or compelling factors, without another cardiac or systemic cause of LVH),	n=102 (50 female); 80 phenotype-positive<br><br>51±16 years	-Previous myocardial infarction<br>>50% stenosis<br>-Previous PCI or CABG<br>-Stroke<br>-Aortic valve stenosis<br>-Previous SRT<br>-CKD<br>-eGFR<30	-Questionnaire<br>-Physical<br>-Cardiac troponin<br>-ECG<br>-ECHO<br>-CMR
Wasserstrum et al. 2019 [22]	Pre-post	-≥12 years<br><br>-HCM (LV thickness ≥ 15 mm, without	n=45 (14 female)<br><br>58±13	-Inconsistent diagnosis,<br>-inability to afford participation<br>In the rehabilitation	-Data on EST, ECHO, subjective wellbeing, and adverse events

		evidence of pathophysiology that might cause secondary myocardial hypertrophy<br><br>-any clinical indication for referral to cardiac rehabilitation<br><br>-participation in the cardiac rehab. program for ≥3 consecutive months<br><br>-Availability of Exercise test data		program	during exercise
Sweeting et al. 2018. [23]	Pre-post	-18-60 years<br><br>-definite diagnosis of HCM, - sufficient English to complete the survey and interview, access to a mobile phone<br><br>-participants who answered "no" to the screening question "Are you active for at least 30 minutes on at least 5 days of the week?"	n=25<br>(10 female)<br><br>42±13<br>years	-major co-morbidities<br>-cases where participation was deemed unsafe by the cardiologist	A survey (comprised of IPAQ, SF-36v2, barriers to physical activity and self-efficacy scale), accelerometer, HR monitor
Perez Sanchez et al. 2018 [24]a	Cohort	-genotyped HCM families with mutations in *MYBPC3* and *MHY7*<br><br>- HCM defined as unexplained LVH in probands (≥15mm without other cardiac or systemic causes of LVH), relatives considered affected in the context of familial HCM	n=272<br>(117 female)<br><br>(192 with HCM diagnosis)<br><br>49 ± 17 years	Relatives of patients with HCM who did not carry the mutation	Physical, ECHO, questionnaire
Dejgaard et al. 2018 [25][a]	Cross sectional	-Unrelated HCM index patients (HCM LVH +)<br><br>- Gt+ LVH- patients	n=187<br>(89 female);<br><br>121 phenotype-positive<br>(47 female)<br><br>49±16 years	-Previous myectomy<br>-Alcohol septal ablation	-Questionnaire<br>-ECHO<br>-CMR<br>-Medical records data<br>-Genetic testing
Saberi et al. 2017 [26][a]	RCT	- ≥18 years<br><br>-HCM (unexplained LVH >13 mm in any wall segment)	n=136<br>(57 female)<br><br>Exercise (n=67): 50.5±13.2 years<br><br>Usual activity (n=30): 50.0±13.5 years	-History of exercise-induced syncope or VA<br>-Medical refractory LVOT obstruction evaluated for SRT<br>-Planned ICD implantation<br>-Less than 3 months from ICD implantation or SRT<br>-Hypotensive response during exercise	Baseline and after 16 weeks:<br>- questionnaire<br>-ECG<br>-biomarkers<br>-ECHO<br>-CPET<br>-CMR<br>-QoL

				-NYHA IV or CCS IV in the previous 3 months<br>-LVEF<55%<br>-Life expectancy <12 months<br>-Pregnant/planned pregnancy<br>-Noncardiac exercise limitations<br>-Unwilling to refrain from HI or competitive sports	
Klempfner et al. 2015 [27]	Pre-post	-diagnosis of HCM per ACCF/AHA and ESC position statements<br><br>-adult symptomatic HCM patients despite optimal therapy, NYHA II-III<br>-stable condition in the past 6 months<br><br>-physical capability to undertake an exercise program	n=20<br>(6 female)<br><br>62±13 years	-history of exercise-induced syncope, uncontrolled ventricular arrhythmia, NYHA IV, HF, or severe angina (CCS III–IV), and non-cardiac causes of exercise limitations.	-physical<br>-24h ECG Holter<br>-ECHO<br>- EST

Abbreviations: CABG=coronary artery bypass graft; CAD=coronary artery disease; CCS=Canadian Cardiovascular Society; CIED=cardiac implantable electronic device; CKD=chronic kidney disease; CPET=cardiopulmonary exercise test; CVD=cardiovascular disease; ECG=electrocardiogram; ECHO=echocardiography; eGFR=estimated Glomerular Filtration Rate; EST=exercise stress test; HCM=hypertrophic cardiomyopathy; HF=heart failure; HT=hypertension; ICD, implantable Cardioverter-Defibrillator; LGE=late gadolinium enhancement; LVEF=left ventricular ejection fraction; LVOT=left ventricular outflow tract; LVWT=maximal left ventricular wall thickness; NYHA=New York Heart Association; N/A=not applicable; PA=physical activity; PCI=percutaneous coronary intervention; QOL=quality of life; RCT=randomized controlled trial; RM=repetition maximum; RPE=rating of perceived exertion; RT=resistance training; SRT=septal reduction therapy; VA=ventricular arrhythmia.

Study	Selection	Comparability	Outcome		**Score**

	Representativeness of the sample	Selection of the non-exposed cohort	Ascertainment of exposure	Outcome of interest was not present at start of study	Based on design and analysis	Assessment of outcome	Follow-up	Adequacy of follow-up	
Cavigli et al. 2024 [17]	+	+	+	+	0	+	+	0	**6**
Lampert et al. 2023 [18]	+	+	0	+	+	+	+	+	**7**
Perez Sanchez et al. 2018 [24]	+	+	0	0	++	+	0	0	**5**

Table S4. Newcastle-Ottawa quality assessment scale for cohort studies

Studies are assessed with a maximum of nine points. A higher score indicates better study quality. Good (7-9 points), fair (5-6 points), and poor/unsatisfactory (0-4 points).

Table S5. Newcastle-Ottawa quality assessment scale for cross sectional studies

Study	Selection				Comparability	Outcome		**Score**
	Representativeness of the sample	Sample size	Non-respondents	Ascertainment of exposure	Based on design and analysis	Assessment of outcome	Statistical test	
Hassanzada et al. 2024 [16]	+	0	0	++	++	+++	+	**9**
Kwon et al. 2021 [20]	+	+	0	++	+	+++	+	**9**
Aengevaeren et al. 2019 [21]	+	0	0	++	0	+++	+	**7**
Dejgaard et al. 2018 [25]	+	0	+	+	++	++	+	**8**

Studies are assessed with a maximum of nine points. A higher score indicates better study quality. Good (7-9 points), fair (5-6 points), and poor/unsatisfactory (0-4 points).

Table S6. Quality assessment using NIH Quality assessment Tool for Before-After (Pre-Post) studies with no control group

Criteria	Klempfner et al. 2015 [27]	Wasserstrum et al. 2019 [22]	Sweeting et al. 2018. [23]
1. Was the study question or objective clearly stated?	Y	Y	Y
2. Were eligibility/selection criteria for the study population prespecified and clearly described?	Y	Y	Y
3. Were the participants in the study representative of those who would be eligible for the test/service/intervention in the general or clinical population of interest?	N	N	N
4. Were all eligible participants that met the prespecified entry criteria enrolled?	NR	N	N
5. Was the sample size sufficiently large to provide confidence in the findings?	N	N	N
6. Was the test/service/intervention clearly described and delivered consistently across the study population?	Y	Y	Y
7. Were the outcome measures prespecified, clearly defined, valid, reliable, and assessed consistently across all study participants?	Y	Y	Y
8. Were the people assessing the outcomes blinded to the participants' exposures/interventions?	NR	NR	NR
9. Was the loss to follow-up after baseline 20% or less? Were those lost to follow-up accounted for in the analysis?	NA	N	N
10. Did the statistical methods examine changes in outcome measures from before to after the intervention? Were statistical tests done that provided p values for the pre-to-post changes?	Y	Y	Y
11. Were outcome measures of interest taken multiple times before the intervention and multiple times after the intervention (i.e., did they use an interrupted time-series design)?	N	N	N
12. If the intervention was conducted at a group level (e.g., a whole hospital, a community, etc.) did the statistical analysis take into account the use of individual-level data to determine effects at the group level?	NA	NA	NA

Y, yes; N, no; NA, not applicable; NR, not reported

Overall Quality: Fair

Table S7. Comorbid conditions of HCM patients in the interventional studies

Author, publication	Study	Participant	Hypertens	Coronar	TIA or	Diabetes	Hypercholes	Lung	Renal

year	type	number	ion	y artery disease	Stroke		terolemia	disease	failure
Basu et al. 2025 [14][a]	RCT	n=80	26 (32.5%)	N/A	1 (1.3%)	3 (3.8%)	19 (23.8%)	N/A	2 (2.5%)
Gudmundsdottir et al. 2025 [15][a]	RCT	n=59	25 (42.4%)	9 (15.3%)	4 (6.8%)	4 (6.8%)	N/A	2 (3.4%)	/
Cavigli et al. 2024 [17][a]	Cohort	n=71	N/A	N/A	N/A	N/A	N/A	N/A	N/A
Lampert et al. 2023 [18][a]	Cohort	n= 1660	N/A	N/A	N/A	N/A	N/A	N/A	N/A
Mac Namara et al. 2023 [19][a]	RCT	n=22	N/A	N/A	N/A	N/A	N/A	N/A	N/A
Wasserstrum et al. 2019 [22]	Pre-post	n=45	24 (53.3%)	11 (24.4%)	5 (11%)	6 (13.3%)	N/A	7 (15.5%)	N/A
Sweeting et al. 2018 [23]	Pre-post	n=25	N/A	N/A	N/A	N/A	N/A	N/A	N/A
Saberi et al. 2017 [26][a]	RCT	n=136	30 (22%)	9 (6.6%)	N/A	9 (6.6%)	N/A	4 (2.9%)	N/A
Klempfner et. al 2015 [27]	Pre-post	n=20	8 (40%)	1 (5%)	2 (10%)	2 (10%)	N/A	1 (5%)	1 (5%)

[a]Studies included in the quantitative analysis.

Abbreviations: HCM=hypertrophic cardiomyopathy; N/A not applicable; RCT=randomized controlled trial; TIA=transient ischemic attack;

Figure S1. Risk of bias assessment for randomized controlled trials

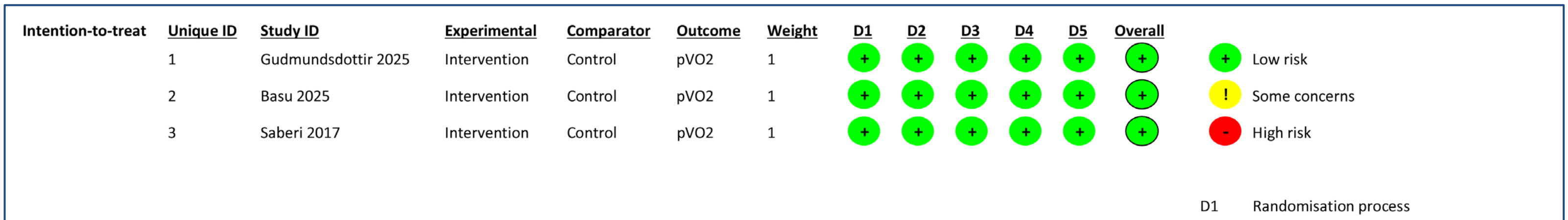

Fig. S2. Sensitivity analysis for the difference in the occurrence of AF between the MIPA and the sedentary control group.

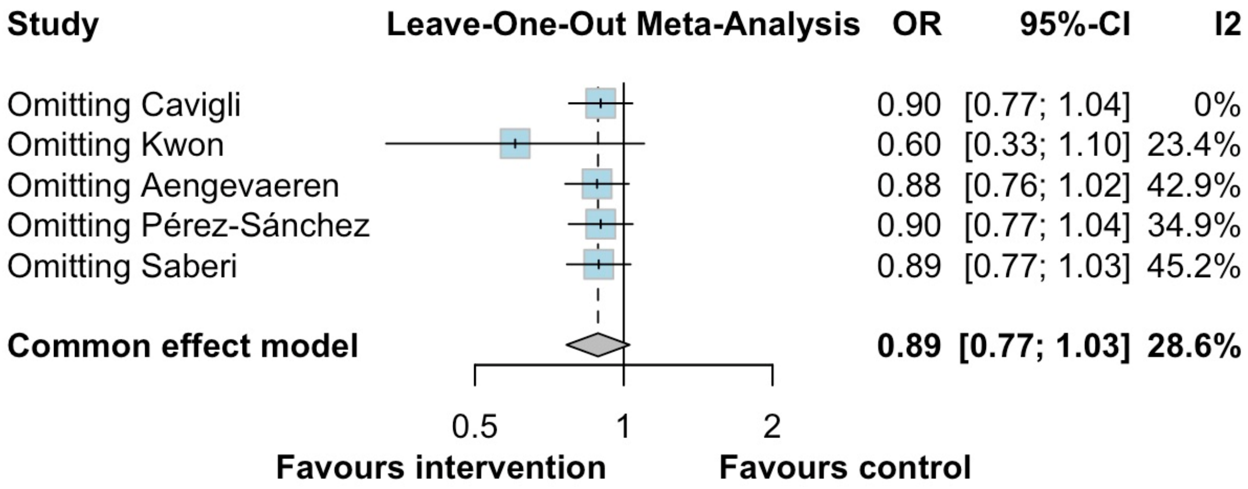

AF=atrial fibrillation. MIPA=moderate-intensity physical activity; OR=Odds ratio; CI=Confidence Interval.

Fig. S3. Sensitivity analysis for the difference in the occurrence of NSVT between the MIPA and the sedentary control group.

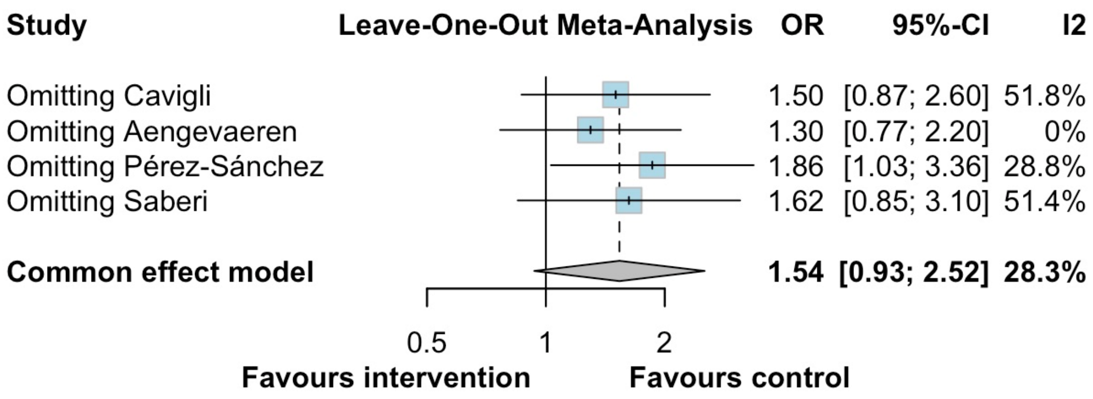

MIPA=moderate-intensity physical activity; NSVT=non-sustained ventricular tachycardia. OR=Odds ratio; CI=Confidence Interval.

Fig. S4. Sensitivity analysis for the difference in the occurrence of syncope between MIPA and sedentary control group.

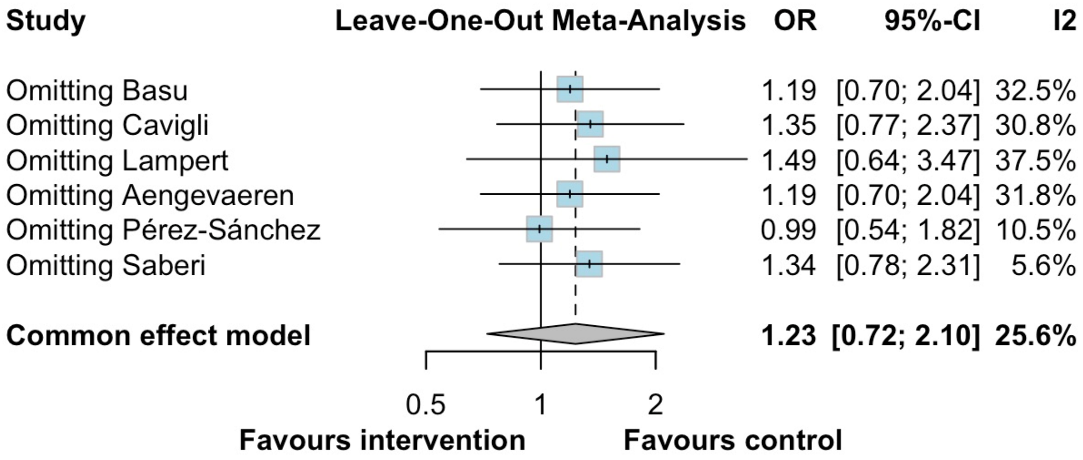

MIPA=moderate-intensity physical activity. OR=Odds ratio; CI=Confidence Interval.
